# Genetic monogamy and mate choice in a pair-living primate

**DOI:** 10.1038/s41598-020-77132-9

**Published:** 2020-11-23

**Authors:** Sofya Dolotovskaya, Christian Roos, Eckhard W. Heymann

**Affiliations:** 1 Behavioral Ecology and Sociobiology Unit, German Primate Center, Göttingen, Germany; 2 Primate Genetics Laboratory, German Primate Center, Göttingen, Germany; 3 Gene Bank of Primates, German Primate Center, Göttingen, Germany

**Keywords:** Behavioural genetics, Ecological genetics

## Abstract

In pair-living mammals, genetic monogamy is extremely rare. One possible reason is that in socially monogamous animals, mate choice can be severely constrained, increasing the risk of inbreeding or pairing with an incompatible or low-quality partner. To escape these constraints, individuals might engage in extra-pair copulations. Alternatively, inbreeding can be avoided by dispersal. However, little is known about the interactions between mating system, mate choice, and dispersal in pair-living mammals. Here we genotyped 41 wild individuals from 14 groups of coppery titi monkeys (*Plecturocebus cupreus*) in Peruvian Amazon using 18 microsatellite loci. Parentage analyses of 18 young revealed no cases of extra-pair paternity, indicating that the study population is mostly genetically monogamous. We did not find evidence for relatedness- or heterozygosity-based mate choice. Despite the lack of evidence for active inbreeding avoidance via mate choice, mating partners were on average not related. We further found that dispersal was not sex-biased, with both sexes dispersing opportunistically over varying distances. Our findings suggest that even opportunistic dispersal, as long as it is not constrained, can generate sufficient genetic diversity to prevent inbreeding. This, in turn, can render active inbreeding avoidance via mate choice and extra-pair copulations less necessary, helping to maintain genetic monogamy.

## Introduction

Since sexual selection in both males and females is influenced by the number of mating partners, extra-pair paternities (EPP) play an important role in the evolution of mating systems^1,2^. EPP are common in pair-living, or socially monogamous birds and mammals (see Table 1 for definitions used in this study), including humans, while genetic monogamy is a very rare phenomenon^1,3,4^. Among pair-living mammals — which constitute up to 9% of mammal species, depending on the classification method^5,6^ — strict genetic monogamy (no cases of EPP) has been reported for only seven species so far (Table 2). Four other species can be considered as “mostly” genetically monogamous, with the rate of EPP < 10%. However, for most pair-living mammal species, genetic paternity data simply does not exist yet, and therefore our understanding of the frequency of genetic monogamy is very incomplete.

**Table 1:** Terminology used in this study, proposed in^7^ and based on the framework of Kappeler^8^.

**Pair-living** (who lives with whom)*:* a type of social organization where one adult male and one adult female share a home range, possibly with their non-reproducing offspring. This is often referred to as “social monogamy”					
**Pair-bonded** (who is affiliated with whom): a type of social structure where one adult male and one adult female have an affiliative relationship to the exclusion of other adults, as evidenced by behavioral, emotional, and endocrinological characteristics					
**Sexual monogamy** (who mates with whom): a type of social mating system where one adult male and one adult female have an exclusive mating relationship during at least one reproductive season					
**Genetic monogamy** (who produces offspring with whom): a type of genetic mating system where one adult male and one adult female produce offspring exclusively with each other over a set of multiple births (at least one reproductive season for species that produce more than one infant per litter and over more than one consecutive reproductive seasons for species with singleton births)					
**Biparental care** (who provides parental care): a type of care system where a mother and putative father regularly provide offspring care

**Table 2:** List of genetically monogamous mammals with no extra-pair paternities (EPP) detected. Also included two predominantly genetically monogamous species with the rate of EPP < 10%.

Order	Species	Sample size	Genotyping method used	N of EPP cases found	References
Primates	Azara’s owl monkey (*Aotus azarae*)	35 offspring of 29 family groups (128 animals in total)	14 microsatellite loci	0	^3^
Primates	Bornean gibbon (*Hylobates muelleri*)	4 offspring of 4 family groups (13 animals in total)	16 microsatellite loci	0	^9^
Artiodactyla	Kirk’s dik-dik (*Madoqua kirkii*)	12 offspring of 11 family groups (68 animals in total)	7 microsatellite loci	0	^10^
Carnivora	Coyotes (*Canis latrans*)	96 offspring of 18 family groups (236 animals in total)	12 microsatellite loci	0	^11^
Rodentia	California mouse (*Peromyscus californicus*)	82 offspring of 22 complete groups, plus 17 offspring from incomplete groups (samples from father or mother not available)	DNA fingerprinting using restriction enzyme	0	^12^
Rodentia	Malagasy giant jumping rat (*Hypogeomys antimena*)	60 offspring of 28 family groups (139 animals in total)	Polymorphisms of a major histocompatibility complex class II gene DQA using sequencing and single-strand conformation polymorphism analysis	0, with 3 cases of male and 3 cases of female replacement but no litters sired by multiple fathers	^13^
Rodentia	Taiwan vole (*Microtus kikuchii*)	31 offspring of 20 family groups	10 microsatellite loci	0, with 2 cases of female replacement but no litters sired by multiple fathers	^14^
Rodentia	Eurasian beaver (*Castor fiber*)	(a) 18 offspring of 9 colonies plus 6 family groups with only adults (38 animals in total)^15^; (b) 166 offspring of 48 family groups (356 animals in total)	(a) 15 microsatellite loci; (b) 27 single nucleotide polymorphisms	(a) 0, with one possible female replacement but no litters sired by multiple fathers; (b) 9, corresponding to the EPP rate of 5.4%; 7 offspring were sired by neighboring males, in 2 cases the paternity could not be assigned	(a) ^15^; (b) ^16^
Primates	Indri (*Indri indri*)	12 offspring of 7 family groups (26 animals in total)	6 microsatellite loci	1, corresponding to the EPP rate of 8.3%; social father excluded as genetic father but no other male indicated as likely father	^17^
Primates	﻿Golden-cheeked gibbons (*Nomascus gabriellae*)	10 offspring of 6 family groups (29 animals in total)	8 microsatellite loci	1, corresponding to the EPP rate of 10%; a lone non-territorial male confirmed as genetic father	^18^
Primates	White-handed gibbons (*Hylobates lar*)	41 offspring, 27 born in pair-living groups and 15 born in multi-male groups (89 animals in total)	12 microsatellite loci	3, corresponding to the EPP rate of 7.3%; 2 were sired by neighboring males, in 1 case paternity could not be assigned	^19^

Rates of EPP vary substantially between species and populations and have been shown to be affected by various factors, such as, for example, intensity of male care, pair-bond strength, and population density^3,4,20,21^. The intriguing question is why some individuals engage in mating with multiple partners while others do not. The advantages to males of engaging in extra-pair copulations (EPC) are well recognized, as males are expected to increase their fitness by increasing the number of mating partners as the result of their higher potential reproductive rate^2,22^. However, in pair-living species with biparental care, potential reproductive rates and, consequently, levels of intra-sexual competition will be more similar for males and females^2^. As a result, both sexes might be expected to gain benefits from engaging in EPC^23^. These benefits might include insurance against social partner’s infertility, maximizing genetic diversity among offspring, or increasing offspring genetic quality by mating with individuals that are more genetically compatible or of higher genetic quality (reviewed in, e.g.,^24^).

One potential advantage of EPC to both sexes could be related to limitations in mate choice. In pair-living species with biparental care, especially in those with low mobility and low breeding density, mate choice can be highly constrained. First, mates become unavailable once paired. And second, individuals may face a conflict between choice for direct benefits (territory quality, intensity of parental care) and indirect genetic benefits (partner genetic quality or compatibility). As a result of this constrained mate choice, individuals may end up paired to a genetically incompatible, closely related, or low-quality partner. To escape these constraints, animals might seek EPC that would allow them to gain indirect benefits while still taking advantage of direct benefits provided by the social partner^23^. This strategy has been demonstrated in various bird species^20,25^. In mammals, the evidence is much more limited. In Alpine marmots, *Marmota marmota,* and meerkats, *Suricata suricatta*, EPP rates were found to be higher in pairs where partners were more closely related^26,27^. But, to our knowledge, the positive relationship between partners’ genetic similarity and EPP rates has been only demonstrated in one pair-living mammal species: in fat-tailed dwarf lemur, *Cheirogaleus medius*, females sharing more major histocompatibility complex (MHC)-supertypes with their social partner were shown to engage in more EPC^28^.

Any mate choice, whether it is a choice for social or extra-pair partner, is expected to maximize not only direct fitness benefits, but also indirect (genetic) benefits, expressed as increased genetic quality of offspring. The closely related hypotheses of genetic compatibility and heterozygosity posit that individuals benefit from choosing a mate that will maximize offspring heterozygosity^29−31^. Thus, animals are expected to choose ﻿mates that are genetically unrelated or dissimilar at some fitness-related genes (e.g., MHC genes). An increase in offspring heterozygosity resulting from this disassortative mating is expected to increase offspring fitness, as indicated by links between individual heterozygosity and various fitness proxies, such as survival, reproductive success, and parasite resistance (e.g.,^25,32,33^; reviewed in^29^). In addition, irrespective of genetic compatibility, individuals might also benefit from choosing genetically higher-quality mates, i.e., those who carry “good genes” or are more heterozygous^29^. With regard to heterozygosity, two opposing hypotheses have been suggested ^29^. First, heterozygosity may be expected to be positively correlated between mates, because less heterozygous individuals might be less successful in finding the best, i.e., more heterozygous partners. Alternatively, the correlation of heterozygosity between partners might be negative, because less heterozygous individuals need to compensate for their lower quality by choosing more heterozygous mates. Additionally, as heterozygous partners are expected to have higher fitness, they should be more likely to provide direct benefits such as increased parental care, fertility or superior territory^29,34^.

Mate choice based on heterozygosity was demonstrated in various species of birds and mammals. For example, in blue tits, *Cyanistes caeruleus*, heterozygosity was positively correlated between social mates, indicating that mating preferences were based on partner’s heterozygosity^35^. In Antarctic fur seals, *Arctocephalus gazella*, where females exert choice by moving across a breeding colony to visit largely stationary males, females were shown to move further to maximize the balance between male high heterozygosity and low relatedness^34^. In many species, mate choice was shown to be based on MHC loci dissimilarity (e.g., fat-tailed dwarf lemur^28^) or, conversely, similarity (probably an adaptation to local pathogens, shown in, e.g., Malagasy giant jumping rat, *Hypogeomys antimena*, and European badgers, *Meles meles*^36,37^). Finally, relatedness-based mate choice, while demonstrated in some species, such as Antarctic fur seals, was not found in many other studied species, such as fat-tailed dwarf lemurs, blue tits, and great reed warblers, *Acrocephalus arundinaceus*^28,35,36,38^.

The absence of relatedness-based mate choice in pair-living species, such as fat-tailed dwarf lemurs or blue tits, might seem particularly surprising, because the risk of inbreeding in such species is expected to be high due to constrained mate choice. In the absence of other options, or as a result of trade-offs between a choice for a good territory vs. a choice for unrelated/compatible partner, individuals might end up paired with close relatives. Therefore, pair-living animals may be expected to use relatedness-based mate choice to “actively” avoid pairings with closely related individuals^39^. However, pairings with close relatives might be also avoided “passively” by natal dispersal that can disrupt the associations of opposite-sex kin and thus prevent matings between them^40^. Dispersal was shown to be sufficient to avoid inbreeding or reach a certain level of genetic dissimilarity in many situations^35,38,41^. It remains unclear, however, if dispersal has to be sex-biased to generate enough local genetic dissimilarity between breeding females and males to avoid inbreeding. In most mammals, males are the dispersing sex, because in polygynous mating systems, which are prevailing in mammals, males experience stronger intra-sexual competition for mates than females^40,42^. Following the same logic, mammals that mate monogamously or cooperatively with high levels of reproductive monopolization by a dominant pair are expected to have little or no sex bias in dispersal. This was found to be true in some mammals, such as the genetically monogamous Azara’s owl monkey, *Aotus azarae*, where both sexes disperse, or cooperatively breeding meerkats, where dispersal is only slightly male-biased^43,44^. However, in other mammals, e.g., genetically monogamous California mice, *Peromyscus californicus*, or socially monogamous greater white-toothed shrew, *Crocidura russula*, dispersal was found to be female-biased^45,46^.

Here, we present a comprehensive study of the genetic mating system, mate choice, and dispersal in a wild population of coppery titi monkeys, *Plecturocebus cupreus.* Titi monkeys (genera *Callicebus*, *Plecturocebus*, and *Cheracebus*) exhibit almost all the elements of the “monogamy package”, such as pair living, strong long-term pair bonds, an exceptionally high level of male care (the infant is carried almost exclusively by the social father), territoriality, and sexual monomorphism^47−50^. The only missing component which has yet to be characterized is the genetic mating system. Titis are one of the very few mammalian taxa that exhibit both high level of male care and strong pair bonds, two characteristics shown to affect the rates of EPP in mammals^3^. The examination of their mating system and the proximate mechanisms of its maintenance may, therefore, shed light on the evolution of social and genetic monogamy in mammals. In this study, we first examined the mating system of coppery titis using a set of 18 newly developed microsatellite loci that can be universally applied to Neotropical monkeys. Second, we tested for evidence of relatedness- and/or heterozygosity-based mate choice. Finally, to see if dispersal is sex-biased, we compared genetic relatedness and diversity patterns in adult females and males and performed spatial genetic analysis. Given consistent pair living, strong pair bonds, and high levels of male care in coppery titis, we predicted them to be genetically monogamous or have a very low rate of EPP. Since the risk of inbreeding is expected to be especially high for long-lived pair-living species such as titis, we expected to find evidence for active inbreeding avoidance via mate choice and/or for heterozygosity-based mate choice. We predicted both sexes to disperse, as expected from a pair-living territorial mammal with biparental care.

## Results

### Are titis genetically monogamous?

**Figure 1:**
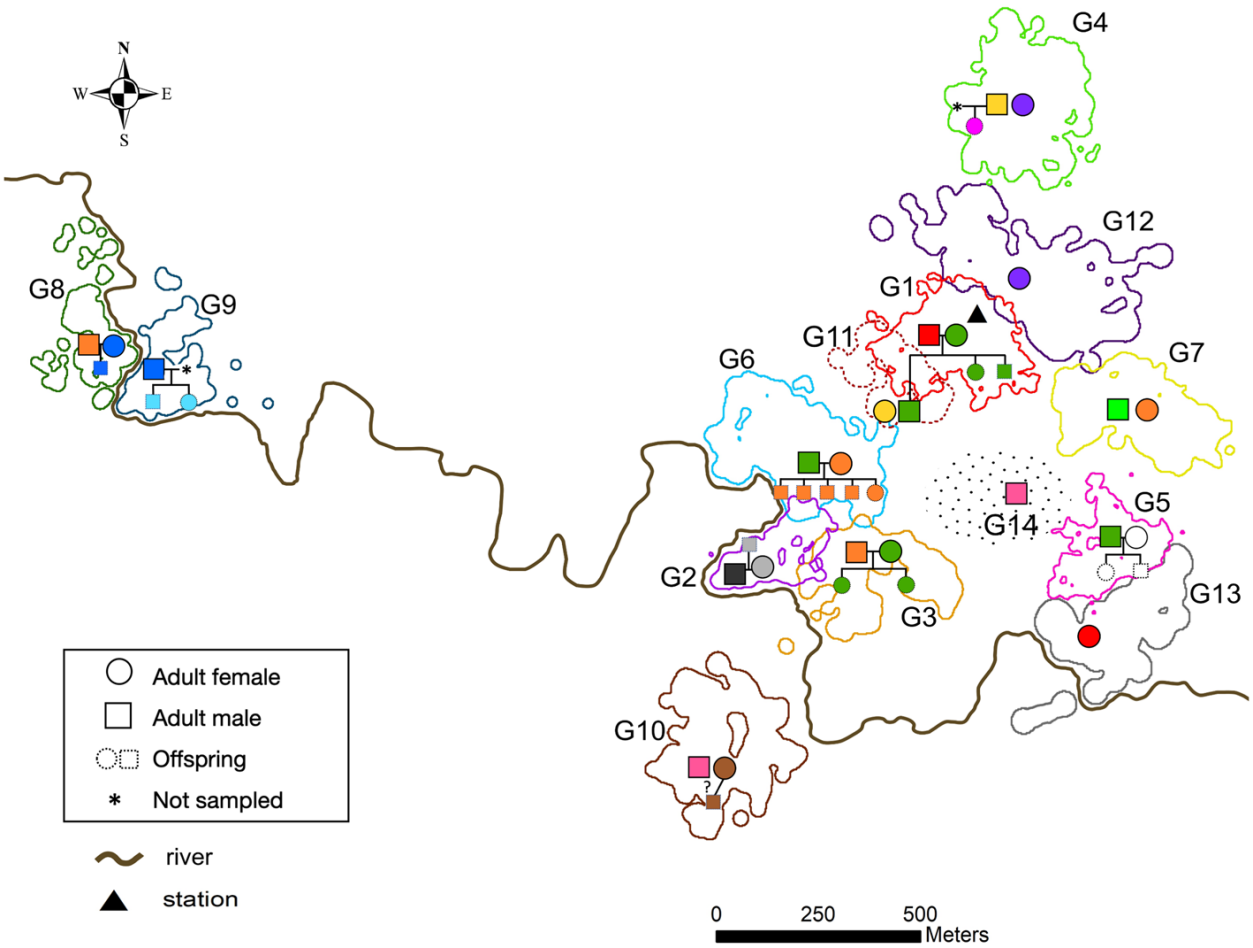
Home ranges, mtDNA haplotypes, and parentage for sampled individuals within study groups. Circles and squares with continuous outline represent adult females and males, respectively; smaller circles and squares with dotted outline represent female and male offspring, respectively. The colors of circles and squares represent different mtDNA haplotypes. Home ranges of study groups were estimated using the 95% fixed kernel density method with ArcGIS Desktop 10.6 (ESRI; https://desktop.arcgis.com) (see more details in “Methods”). The home range of Group 14 is depicted as dotted ellipse because we did not have enough GPS data to reliably estimate its home range. The home range of Group 11 is depicted as dotted line because this newly established territory was most likely not permanent and bound to shift later (see Supplementary Materials for details of the dispersal event and Methods for more details on the habitat). The map was created in ArcGIS Desktop 10.6 and modified with Inkscape 1.0.1 (https://inkscape.org/).

Our analyses did not indicate any cases of EPP. In all cases of assigned paternity (17 offspring in 9 social groups, 1 to 5 offspring per group; Fig. 1, Supplementary Table S1), social fathers were identified as genetic fathers of all offspring in their respective family groups. In one case, paternity remained unassigned. The juvenile male from Group 10 had three mismatches with the adult male of the group, and ﻿Delta score calculated by Cervus (the difference between the likelihood ratios for two most likely candidate parents) was zero, indicating both this male and the adult male of Group 8 as most likely fathers. At one of the loci with mismatches (chr09a), the juvenile was homozygous, likely resulting from allelic dropout or genotyping error, at two other loci (chr07a, chr08a), the juvenile was heterozygous, so we can only suggest that it was a result of a genotyping error. Unfortunately, this juvenile had the minimum number of typed loci among all the sampled animals and also was the only individual in our dataset for whom we only had one fecal sample collected. Therefore, we could not control for the errors using another sample like we did for all other individuals.

Apart from the three allelic mismatches in the case of the unassigned paternity, we found only two more cases of mismatches (Supplementary Table S1). Father-daughter dyad from Group 1 had a mismatch at locus chr10a. Since the daughter was homozygous at this locus, this was most likely a result of allelic dropout or genotyping error. Father-daughter dyad from Group 9 had a mismatch at locus chrXa; the daughter was not homozygous at this locus but considering the high likelihood of parentage given by the other loci, we assumed that this mismatch was due to a genotyping error.

Of eight sampled social mothers, seven were identified as genetic mothers of all offspring in their family groups (17 offspring in 7 groups, 1 to 5 offspring per group). One inferred case of female replacement was detected, as the adult female of Group 4 was not identified as the genetic mother of the group’s juvenile offspring; they did not share the mtDNA haplotype and had 11 allelic mismatches. No other female in our sample was identified as the most likely mother for this offspring or shared a mtDNA haplotype with it. The social father of this juvenile was indicated as the genetic father.

All assignments were made with a 95% confidence level in Cervus software and confirmed with Colony software (Supplementary Table S1). The assignments did not change when the set of known mother–offspring pairs was excluded from the priors. Colony also yielded strong support for full-sib relationships between all offspring from the same groups, confirming correct parentage assignments.

### Is mate choice based on relatedness or heterozygosity?

We found no evidence for relatedness-based mate choice. There was no difference between relatedness of real mating pairs and randomly generated mating pairs (Li's relatedness estimators (see “Methods”): − 0.048 vs. − 0.021, p = 0.565; n = 10 pairs, breeding pool of 12 females and 12 males). Likewise, we found no evidence for heterozygosity-based mate choice, as homozygosity by loci (HL) was not significantly correlated between pair mates (r = -− 0.527, n = 10 pairs, p = 0.118).

**Figure 2:**
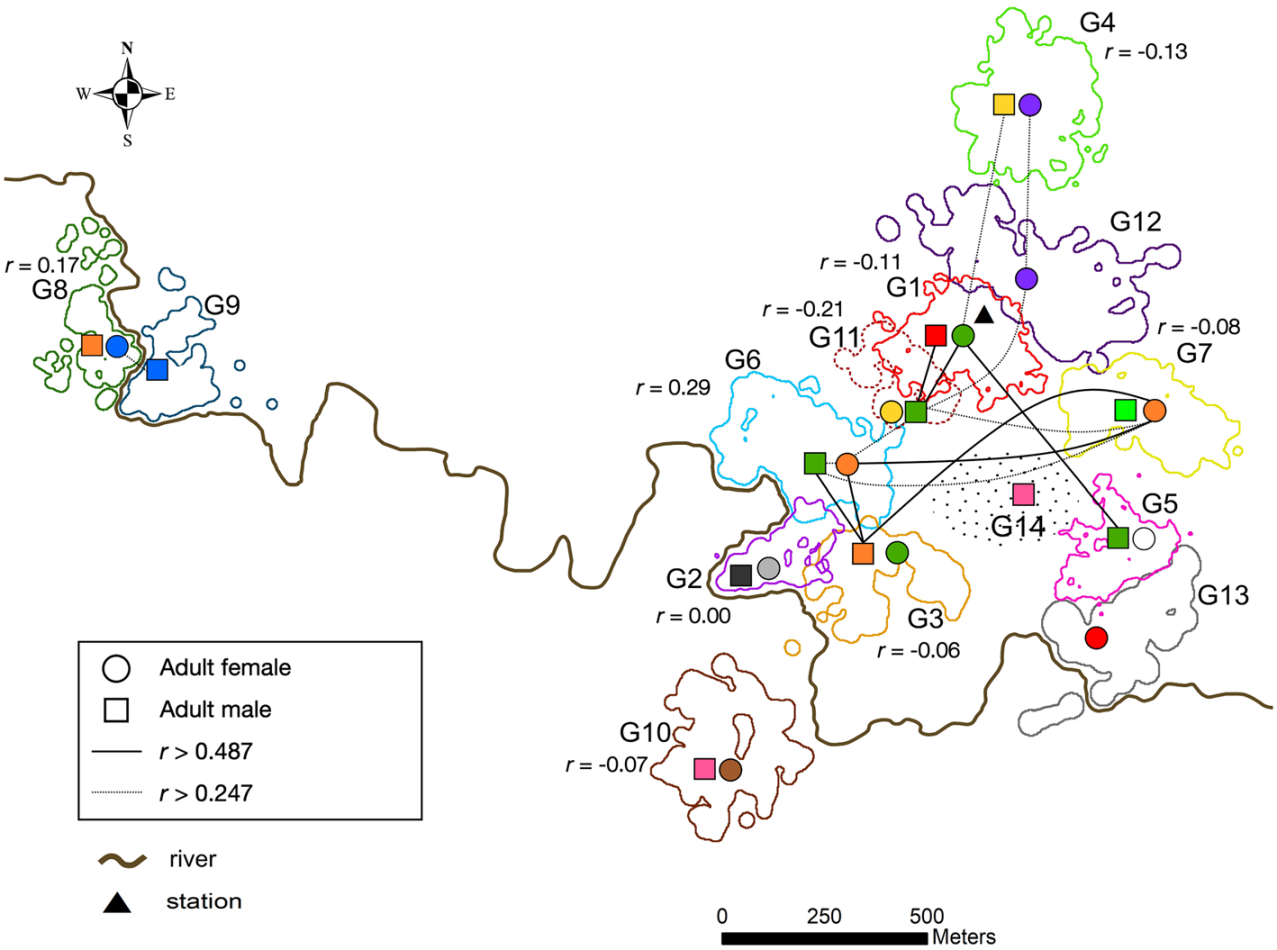
Home ranges, relatedness, and mtDNA haplotypes of adult females (circles) and males (squares) sampled in this study. Home ranges of study groups were estimated using the 95% fixed kernel density method with ArcGIS Desktop 10.6 (ESRI; https://desktop.arcgis.com) (see more details in “Methods”). Relatedness between pair mates (Wang’s relatedness coefficient *r*) is specified for each sampled pair next to the group number. Solid lines connect individuals with Wang’s *r* > 0.487 (mean *r* for simulated parent–offspring dyads), dashed lines connect individuals with Wang’s *r* > 0.247 (mean *r* for simulated half-offspring dyads), individuals with lower *r* are not connected. The map was created in ArcGIS Desktop 10.6 (ESRI; https://desktop.arcgis.com) and modified with Inkscape 1.0.1 (https://inkscape.org/).

Despite the lack of evidence for active inbreeding avoidance via mate choice, relatedness (Wang’s *r*) between mating partners was generally low, averaging -0.033, and none of the pair mates shared the same mtDNA haplotype (Supplementary Table 1, Fig. 1). Only in one pair were the partners found to be related on the level of second-degree kin (Group 6, *r* = 0.285). The mtDNA haplotype network (Fig. 2) showed no clear pattern of haplotype similarity between pair mates: some had closely related haplotypes (e.g., Groups 4, 5, 9), while others had only distantly related haplotypes (e.g., Groups 1, 11).

### Do both sexes disperse and does one sex disperse further than the other?

Our results indicate that both sexes dispersed similar distances. There were no significant differences between adult females and males in mean mtDNA haplotype diversity (0.945 in females, 0.924 in males, permutation test p = 0.766), mtDNA nucleotide diversity (0.027 in females, 0.029 in males, permutation test p = 0.699), mean Wang's relatedness *r* (-0.013 in females, -0.056 in males, mean difference -0.040, lying within the 95% confidence interval (− 0.048–0.054) obtained by bootstrapping), or mean heterozygosity HL (0.184 in females, 0.216 in males, paired t-test p = 0.438).

We did not find evidence for spatial genetic structure in either sex, suggesting that both sexes likely dispersed over varying distances. The correlation between genetic and spatial distances was not significant for either sex, as the 95% CI of autocorrelation *r* values overlapped zero for all distance classes (Supplementary Materials Table S2, Fig. S1). The correlation between mtDNA haplotype distances and spatial distances in females was not significant either (Mantel correlation = 0.048, n = 91 dyads, right-tailed p = 0.342).

## Discussion

The link between mating system, mate choice, and dispersal has rarely been studied in pair-living mammals. Here, we demonstrated that coppery titi monkeys in our study population are mostly genetically monogamous, as we did not find evidence for EPP. We also did not find evidence for relatedness- or heterozygosity-based mate choice. Despite the absence of evidence for active inbreeding avoidance via mate choice, pair mates in our study population had low average relatedness. This finding suggests that natal dispersal can generate sufficient level of genetic dissimilarity between females and males to render both active inbreeding avoidance and EPC less necessary.

Coppery titis are only the second primate species and the seventh pair-living mammal with no evidence of EPP found in a study with an adequate sample size (the study on Bornean gibbon was based on just four infants from four family groups^9^, Table 1). The absence of EPP in titis is not unexpected, as they are consistently pair-living, pair mates spend most of the day within a few meters from each other, sleep together at night and engage in frequent joint visual displays and duetting at the territorial borders^50−54^. This high level of proximity and coordination should make mate guarding easy and effective enough to prevent EPC.

The opportunities to find extra-pair mates are likely limited, too. The home ranges of our study groups have very little overlap (mean 1.4% of pairwise overlap between neighboring groups (0–4.7), unpublished data; Fig. 1), and to find extra-pair mates, individuals would need to intrude into the neighboring home ranges, risking aggression from the same-sex residents. Another way to obtain EPC could be mating with floaters, solitary non-territorial individuals ranging over a wide area after having dispersed from their natal groups. There is accumulating evidence for the importance of floaters in population dynamics of both birds and mammals^55,56^. For example, in Azara’s owl monkeys who are very similar to titis in all aspects of their social system, mated individuals experience intense intra-sexual competition from floaters of both sexes^56,57^. However, the evidence from Azara’s owl monkeys and many other bird and mammal species indicate that floaters do not copulate with the mated animals as often as might be intuitively expected, and EPP are attributed to the neighboring individuals in most cases (e.g.,^1,16,19^; but see^18,58^). In titis, only anecdotal reports of replacements by intruders exist^59,60^, but given the difficulty of detecting floaters, it is possible that they are present in titi populations, too. However, given the high levels of proximity and coordination between pair mates, EPC with the floaters are probably not easier to obtain than EPC with the neighboring individuals. Furthermore, EPC, whether with floaters or neighboring animals, might be costly, with the risks including the higher probability of acquiring sexually transmitted diseases and, for females, the retaliatory withholding of parental care by males^61,62^.

Opportunities for EPC are also affected by population density, with the lower densities making the encounters between individuals and, consequently, EPC less likely^63^. The positive relationship between population density and EPP rates was demonstrated, e.g., in Eurasian beavers, *Castor fiber*, and in many bird species^15,16,21,64^. At our study site, population density was estimated at 34 individuals/km^2^ (unpublished data). This lies within the average range of values reported from behavioral studies for undisturbed populations of titis (26–57 individuals/km^2^); for comparison, reported population density in forest fragments can be as high as 369 individuals/km^2^^48,65,66^. The relatively low density at our study site likely limited the opportunities for EPC. It should be mentioned, however, that for a population of red titi monkeys, *Plecturocebus discolor*, from undisturbed habitat, a preliminary analysis reported three cases of EPP in a sample size of 16 offspring, although these data has not been published yet^67^. The density of this population (57 individuals/km^2^) was slightly higher than that of our study population, the home ranges were on average smaller (7.2 vs. 5.0 ha), and the percentage of home range overlap was larger (1.4% vs. 4.8%)^65^, possibly accounting for the occurrence of EPP.

Although in all cases of assigned paternities the social fathers were identified as genetic fathers for the group offspring (17 offspring born in 9 groups, up to 5 offspring generations per group), we cannot fully exclude the possibility of a low EPP rate in our study population. First, for one juvenile (Group 10), paternity remained unassigned, as neither social father nor any other male from our sample was identified as the most likely father. While this case could be classified as neither extra- nor intra-pair paternity with confidence, it remains possible that this juvenile was sired by an unsampled extra-pair male. In this case, the EPP rate in our study population would be 6%. Alternatively (if we assume that the social father is indeed not the genetic father of the juvenile), this case could be the result of a male replacement in a group. Adult replacements are known to happen in titis, with the breeding positions vacated after the disappearance (presumable deaths) of adults being occupied by same-sex immigrants^47^. Replacements can create groups that do not consist of biological parents and their offspring, leading to the apparent deviations from genetic monogamy even in the absence of EPC. As Group 10 was only habituated shortly before the genetic sample collection and no older offspring were present in it, we could not reconstruct its demographic history. Our data indicates that adult replacements do happen in our study population. The adult female of Group 4 was not identified as the genetic mother of the group’s juvenile offspring, while the adult male was indicated as the genetic father. When we started following this group, the juvenile was estimated to be 7–8 months old based on its body size and the fact that it walked independently (juvenile titis start to walk on their own most of the time at the age of ca. 4.5 months^68^). Lactation in titis lasts ca. 6.5 months^69^, and we did not see the female nursing. Therefore, we assume that the female replacement must have happened within ca. 2 months before we started following the groups, after the juvenile had been weaned.

Second, for the sample size of 17 offspring, an upper limit of 95% confidence interval of EPP level (maximum possible EPP level for a given sample size, assuming no EPP has been found) will be 16.2%. This value is calculated following Brotherton et al.^10^ as 1 − (1 − x)^n^ = y, where x is the maximum possible EPP level, n is the sample size (17), and y is the probability of producing at least one extra-pair offspring (0.95 for 95% confidence). The value of 16.2% is a product of the sample size and does imply that there is 16.2% EPP rate in our study population. To narrow down the confidence interval to at least 5% of EPP, we would need a sample size of 58 offspring, which is difficult to achieve in a reasonable period in a secretive arboreal primate with slow life history, living in pairs and giving singleton births only once a year.

Contrary to our predictions, we did not find evidence for relatedness- or heterozygosity-based mate choice in our study population. Interestingly, despite the absence of evidence for active inbreeding avoidance via mate choice, the pair mates in our study population were on average not related (mean Wang’s *r* = -0.033) and never shared the same mtDNA haplotype (Supplementary Table S1, Fig. 2). Only in one case the pair mates were related on the level of second-degree kin with *r* = 0.285. Low relatedness between mating partners in the absence of active inbreeding avoidance was demonstrated in many other populations of mammals and birds, e.g., grey wolves, *Canis lupus*, arctic foxes, *Vulpes lagopus*, great reed warblers, and blue tits^38,70,71^. In fact, active inbreeding avoidance via mate choice, although demonstrated in some birds and mammals (e.g.,^34,39^), has not been found in most pair-living species^28,35,36,38^; reviewed in^72^. It has been suggested that in most situations, dispersal may be sufficient to avoid inbreeding^38^. By disrupting close-kin associations, dispersal can make the probability of encountering close kin relatively low, rendering active inbreeding avoidance via mate choice unnecessary^72^. In such cases, kin discrimination mechanisms might fail to evolve, and low inbreeding levels that will occasionally occur in such systems will be tolerated^72^.

**Figure 3:**
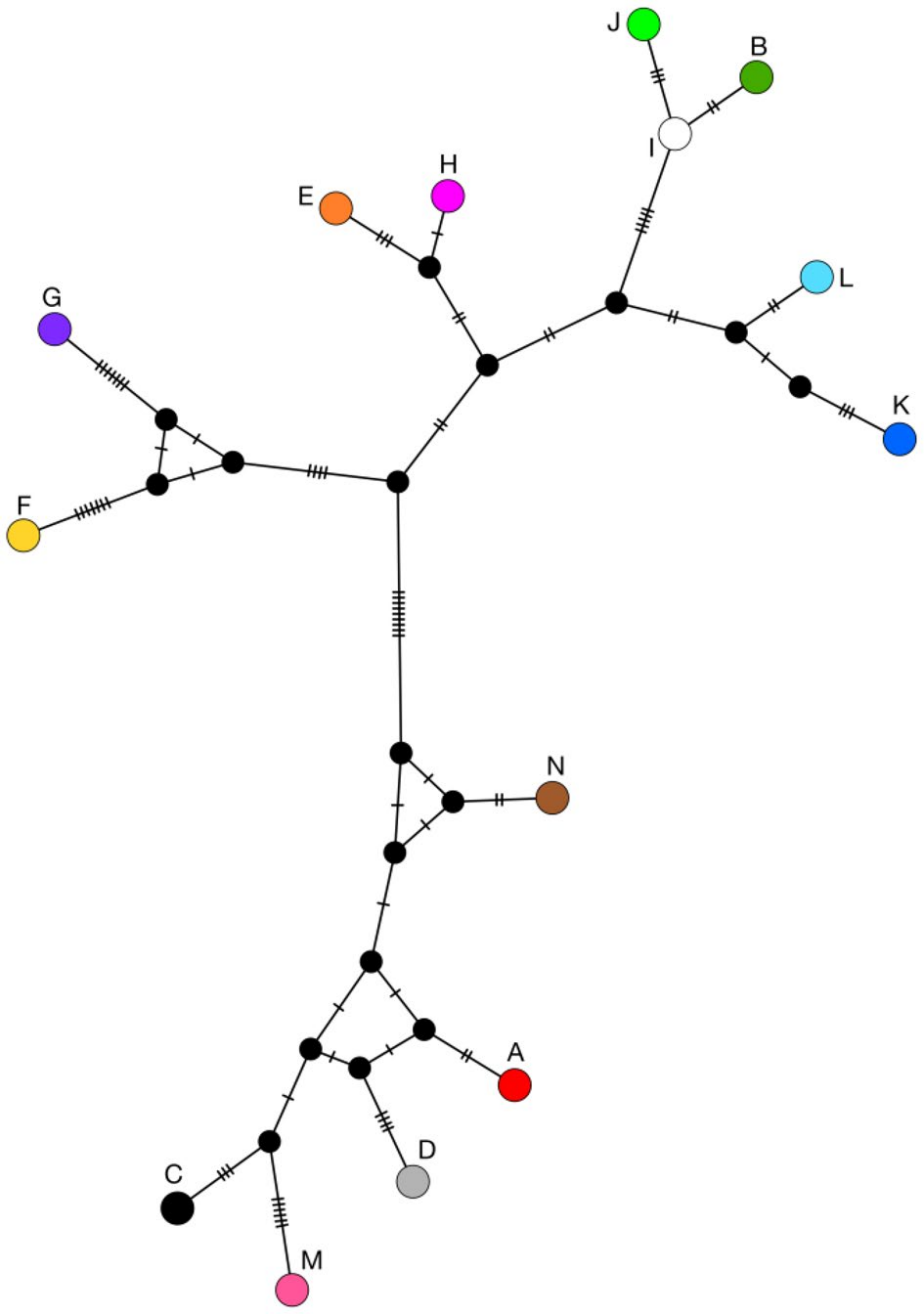
A median joining network of all mtDNA haplotypes found in our study groups, constructed in PopART^73^. The number of hatch marks indicates the number of mutations. Black nodes indicate inferred median vectors. The colors representing mtDNA haplotypes match those used in Fig. 1, 2.

In our study population, dispersal was most likely not sex-biased. This was indicated by the absence of spatial genetic structure in either sex and the lack of obvious geographic clustering in the mtDNA haplotype network (Fig. 2, 3). We found no evidence that dispersal distances differed between sexes, as both mtDNA haplotype diversity and mean relatedness were similar in females and males, suggesting that both sexes migrated opportunistically over varying distances.

As geographic scale of our study was confined, with the maximum distance between home-range centers of only 3200 m, these results should be treated as preliminary. However, they are in line with the direct observation of dispersal and the kin structure of the study population. In one directly observed case of dispersal, a subadult male (the oldest offspring of Group 1), moved to an unoccupied area adjacent to his natal group and later formed a pair (Group 11) with an unknown female (see Supplementary Materials for more details). The female did not have any close relatives among the sampled animals, indicating that she, unlike her mate, had not dispersed from any of the neighboring groups. The closest relative of this female was the adult male of Group 4 with *r* = 0.156 (corresponding to a relatedness level between unrelated and half-sibling), with whom she also shared the same mtDNA haplotype (Supplementary Table S1). Patterns of relatedness between sampled adults (Fig. 1) further suggested that while some dispersers stay in the area (indicated by closely related individuals occupying home ranges that are either adjacent or separated by 1–2 home ranges), others migrate further (as many individuals in the study area have low relatedness). Overall, the absence of evidence for sex bias in dispersal is consistent with the theoretical expectations for the pair-living territorial species, where sexes should experience similar competition for mates and similarly low chances of breeding in acquiring breeding positions in their natal groups^42^.

Our findings on dispersal and mating patterns, although preliminary, are in line with the evidence from other studies indicating that even opportunistic, not sex-biased dispersal can be sufficient to prevent inbreeding, as long as it is unconstrained by habitat fragmentation or other factors. The importance of unconstrained dispersal for inbreeding avoidance was supported in a simulation study based on empirical dataset from golden-crowned sifakas, *Propithecus tattersalli*, showing that high levels of outbreeding can be maintained in a population by a combination of social structure and unconstrained dispersal but without the need for active inbreeding avoidance mechanisms^74^. The link between dispersal and inbreeding risk was further indicated by studies demonstrating a correlation between dispersal distances and inbreeding level (e.g., in great tits, *Parus major*^75^). At our study site, the habitat was undisturbed, and dispersal was most likely unconstrained, ensuring passive inbreeding avoidance. As indicated by one case where pair mates were related on the level of second-degree kin, occasional inbreeding can still occur in such populations and is presumably tolerated.

In addition to dispersal and preferential mating with unrelated individuals, another way to avoid inbreeding is through engaging in EPC. Positive relationship between EPP rates and pair mate relatedness was demonstrated in many bird species^20,23,25^. However, pair-living mammals do not seem to use this strategy often, possibly because mammals are less mobile than birds and it might be harder for them to quickly evade their social mate and sneak EPC. In pair-living mammals, a similar strategy was, to our knowledge, only demonstrated in one species, fat-tailed dwarf lemur, where ﻿females sharing more MHC-supertypes with their social partner engaged in more EPC^28^. In our study population, the absence of evidence for EPP further confirms our suggestion that dispersal in this habitat is unconstrained and the potential for inbreeding is low, rendering EPC less necessary.

Summing up, the current study is the first to examine the link between mating system, mate choice, and dispersal in a wild population of a pair-living primate. We showed that coppery titis in our study population are mostly genetically monogamous. This is likely due to a strong pair bond enabling effective mate guarding and relatively low population density limiting the opportunities for extra-pair copulations. We further showed that coppery titis, despite exhibiting no active inbreeding avoidance via mate choice, still had low relatedness between pair mates. Our results suggest that even opportunistic dispersal, as long as it is not constrained, can create sufficient genetic dissimilarity between opposite sexes to render active mate choice and extra-pair copulations less necessary. Alternatively, the absence of relatedness-based mate choice could be a result of constraints on mate choice, where individuals have so few available mates that they cannot afford to be too selective.

Future studies with larger sample size will be needed to examine the extent of genetic monogamy in this study population, as well as other populations of coppery titis and to further investigate dispersal patterns. In particular, to examine if titis indeed lack the mechanisms for active inbreeding avoidance via kin discrimination, it will be necessary to compare mating patterns and levels of inbreeding in undisturbed vs. fragmented habitats. Additionally, the absence of relatedness- and heterozygosity-based mate choice in our study population, of course, does not mean that mate choice does not occur in titis at any level. To better understand mating patterns in titis, future studies will have to examine if mate choice is based on other factors, such as, e.g., variation in MHC loci, body condition or the size or quality of the territory. Finally, current data do not allow to conclude whether extra-pair paternity is absent (or rare) in this study population because extra-pair copulations are not advantageous to individuals or because socio-ecological constrains prevent them from engaging in extra-pair copulations. To address this question, future studies on larger samples would need to compare genetic quality and fitness of group vs. extra-pair offspring (should there be any) in this or other population of titi monkeys.

## Methods

### Study site and study population

The study was conducted at the Estación Biológica Quebrada Blanco in the north-eastern Peruvian Amazonia (4°21′S, 73°09′W) in June 2017–September 2019. The study area consists mainly of undisturbed primary tropical rainforest of the "terra firme” type (not inundated during the rainy season) interspersed with small swampy areas. The home ranges of Groups 2, 3, and 13 also included land strips of secondary growth along the river that can be inundated for a few days during the height of the rainy season. The spatial gap between Groups 1, 6, 14, and 7 (Figs. 1, 2) is partly occupied by a secondary forest (abandoned buffalo pasture regenerating since 2000) that is avoided by titi monkeys^76^. The spatial gap between Groups 8–9 and the remaining groups (Figs. 1, 2) was a result of study logistics (proximity to camp buildings), not a lack of titi groups in this area.

Study individuals belonged to 14 family groups (Supplementary Table S1, Fig. 1), seven of which (Groups 1–7) were also subject to behavioral studies conducted in June–December 2017 and June–December 2018^51,77,78^. Between the periods of behavioral data collection, the groups were monitored for 2–3 days per month, and genetic samples were collected continuously from the beginning of the study until September 2019. Group 1 had been habituated to the presence of human observers and studied intermittently since 1997; the other groups were habituated during this study. We individually identified all study animals based on the combination of body size, tail shape and colouration, genitalia shape, and natural marks. To control for possible ﻿misidentification of animals in the field, we genotyped study individuals from 2–3 independent samples (only one individual, a juvenile from Group 10, was genotyped from just one sample; see “Results”). We also used a PCR-based sexing assay^79^ to confirm reported sex (and to sex young individuals for whom sex could not be identified in the field).

Home ranges of study groups (Fig. 1, 2) were estimated using the 95% fixed kernel density method with ArcGIS Desktop 10.6 (ESRI) on the basis of GPS points collected during group follows at 10 min intervals with a GPS unit (Garmin GPSMAP 62 s or 64 s; N = 19,456 GPS points, mean per group = 1497 GPS points). Home range sizes varied from 3.6 to 12.98 ha (Supplementary Table S1).

Titis typically give birth to a single infant once a year^47,48,80^. In our study population, most of the births occurred between October and February and only one occurred in June (Supplementary Table S1). As the offspring disperse at the age of 2–4 years^47,48,80^, the pedigree in our study comprised up to 5 generations of offspring per group (Supplementary Table S1).

### Fecal sample collection and DNA extraction

We collected fecal samples from 41 individuals (3–15 samples per individual) living in 14 family groups, including 18 putative offspring of 9 family groups (1 to 5 offspring per group). Five other groups either did not have offspring during the study period (or they had disappeared before we could collect samples) or the samples could not be collected because the offspring were still very young and thus their defecations too diminutive to be detected. Also due to differential habituation to the presence of humans, for some groups we could not obtain samples from all group members. For those groups that were habituated in the beginning of the study period, we collected samples from offspring from several consecutive years.

Fecal samples were collected immediately after an identified individual was seen defecating. We dried the samples in 15 mL falcon tubes containing silica gel beads (Carl Roth, Karlsruhe, Germany) and stored them at ambient temperature, replacing the silica beads when necessary, until shipping to Germany.

We extracted DNA (at least two samples per individual for all animals except one offspring of Group 10; see Results for more details) from ca. 200 mg of feces using the Macherey–Nagel NucleSpin© DNA stool kit with a final elution of the DNA in 50 μL elution buffer. DNA concentration of the extracts was measured using a NanoDrop Spectrophotometer (ND-1000, PEQLAB Biotechnologie GmbH) and a Qubit Fluorometer (Thermo Fisher).

### Microsatellite genotyping

As published microsatellite loci for titi monkeys^81−83^ revealed unreliable results for our study species, we established a new set of 27 di-repeat microsatellite loci that can be universally applied to Neotropical primates (details are described in Supplementary Materials and Supplementary Tables S3–4). To simplify library preparation for genotyping by sequencing, we added adapter nucleotide sequences to the 5′ end of the locus-specific primers.

We amplified all 27 loci in a single multiplex PCR using the Qiagen Multiplex PCR Kit (Qiagen) with a total volume of 25 μL and containing 12.5 μL 2 × Multiplex Master Mix, 1 μL of primer pool (0.2 Mμ of each primer), 1 μL of DNA extract (ca. 200 ng total DNA), and 10.5 μL of RNase-free water. Amplifications were performed with initial denaturation at 95 °C for 15 min, 40 cycles of denaturation at 94 °C for 30 s, annealing at 57 °C for 1.5 min, extension at 72 °C for 1 min, and a final extension at 60 °C for 30 min. PCR products were checked on 1.5% agarose gels together with non-template controls. To prevent false homozygosity due to allelic dropout, we repeated each multiplex reaction three times^84^. In some samples, the total multiplex reaction with all 27 loci yielded low number of sequencing reads; in these cases, we additionally amplified the loci in three separate multiplex reactions with the following primer pools: chr01b–chr07a, chr08a–chr12a, chr12b–chrXa, as this method usually yielded more reads (see Supplementary Materials for details). The reactions and PCR conditions for three separate multiplex reactions were the same as for the total multiplex reaction.

Following amplification, we pooled 5 μL of each multiplex PCR product (or of each PCR product of three separate multiplex reactions), purified the pooled products with the Monarch PCR & DNA Cleanup Kit (New England BioLabs), and quantified them using Qubit Fluorometer (Thermo Fisher). To prepare sequencing libraries, we performed indexing PCRs using Kapa HiFi Hotstart ReadyMix PCR Kit (Roche) with a total volume of 25 μL containing 12.5 μL 2 × Kapa HiFi Hotstart ReadyMix, 1 μL (0.5 Mμ) of each indexing primer (containing individual barcodes) and 100 ng purified PCR product. Indexing PCRs were done with an initial denaturation step at 98 °C for 45 s, followed by 4 cycles of denaturation at 98 °C for 15 s, annealing at 62 °C for 30 s, and extension at 72 °C for 30 s, and a final extension step at 72 °C for 1 min. Full-length libraries were purified with the Monarch PCR & DNA Cleanup Kit (New England BioLabs) and quantified using Qubit Fluorometer (Thermo Fisher). Fragment sizes and molarities were quantified using a Bioanalyzer 2100 (Agilent Technologies). Libraries were diluted to 10 nM and then pooled and sequenced using Miseq Reagent Kit v2 with PhiX DNA (Illumina) added on the MiSeq system (Illumina). Sequencing was performed with 51 forward and 251 reverse read cycles. Only the reverse reads were used for further analysis, while forward reads were only used for MiSeq quality control. To control for laboratory mistakes, we genotyped each sample twice, leading to at least four genotypes per individual.

After sequencing, the samples were demultiplexed using MiSeq Reporter software and then processed using the CHIIMP analysis pipeline^84^. The CHIIMP pipeline calls alleles by first producing unique sequences with﻿ relevant attributes (read counts, sequence length, etc.) for each MiSeq sequence file, querying the sequences for potential PCR artifacts, such as stutter sequences, and then removing all sequences that do not match the locus attributes. All alleles called by CHIIMP were manually checked to validate the results and to correct automated allele calling for those loci that contain “wobble” positions in the primer sequences and are incorrectly processed by CHIIMP. We used a cutoff of 250 reads. Additionally, we accepted alleles if they yielded > 100 reads in at least three genotypes obtained per individual. Alleles with < 100 reads were not called.

Of 27 loci, nine either consistently failed to amplify in our study animals (chr06b, chr11f, chr16b) or proved to be monomorphic (chr02a, chr02b, chr04a, chr10b, chr12a, chr13b) and were excluded from further analysis. The final set consisted of 18 loci, including 17 autosomal and one X-linked locus (chrXa) (Supplementary Table S5). All animals were genotyped at a minimum of 14 loci (16.8 loci on average), and the mean number of alleles per locus was 8.9.

We checked all loci for the presence of null alleles, allelic dropout, and stuttering using Micro-Checker 2.2.5^85^. We assessed Hardy–Weinberg equilibrium (HWE) and calculated observed and expected heterozygosity with PopGenReport 2.2.2^86^. Since the presence of family structure can cause deviations from HWE and bias population genetic analyses, especially in monogamous species, we only included adults in this analysis. The analysis indicated that the population was in HWE. Two loci, chr01b and chr21a, departed from HWE, likely due to the presence of relatives in a study group and/or small sample size.

One of these two loci, chr01b, also showed evidence of null alleles. As the locus did not show any mismatches for the known mother/offspring dyads (see below), we ran all further analyses using two sets of data, one with the full set of loci and another one with locus chr01b excluded. Since the results from these two sets did not differ substantially, we present all further results only for the reduced data set.

### Mitochondrial DNA (mtDNA) genotyping

We genotyped all individuals at the hypervariable region I of the mitochondrial control region using primers 5′-TACCTCGGTCTTGTAAACCG-3′ and 5′-AGGTAGGAACCAGATGCCG-3′, newly designed on the basis of mitochondrial genomes of Neotropical primates available in GenBank. PCR reactions with a total volume of 30 µl contained 1 U BiothermTaq 5000 (Genecraft), 1 × reaction buffer, 0.16 mM of each dNTP, 0.33 µM of each primer, and ca. 100 ng total DNA. PCRs were performed with initial denaturation at 95 °C for 2 min, 40 cycles of denaturation at 95 °C for 1 min, annealing at 58 °C for 1 min, extension at 72 °C for 1 min, and a final extension at 72 °C for 5 min. PCR products were ﻿run on 1% agarose gels, excised from the gel, and then purified with the Monarch DNA Gel Extraction Kit (New England BioLabs) and sequenced on an ABI 3130xL sequencer using both amplification primers and the BigDye Cycle Sequencing Kit (Applied Biosystems). Sequence electropherograms were checked with 4Peaks 1.8 (https://nucleobytes.com/4peaks/index.html) and manually edited and assembled in SeaView 4.5.4^87^; all haplotypes were 567 bp long.

### Statistical analyses

As X-linked loci are haploid in males and cannot be treated in the analyses in the same way as autosomal loci, all the following statistical tests were performed using the set of 16 autosomal loci for both sexes. The data for the X-linked locus chrXa was used separately to manually check for allelic mismatches between candidate parents and offspring in the parentage analyses.

#### (1) Parentage analyses

Parentage was assigned using Cervus 3.0^88^. Cervus compares likelihood ratios of the two most likely candidate parents and assigns parentage based on statistical thresholds generated during the simulation analysis. For Cervus analysis, we used a simulation of 100,000 offspring, an error rate of 0.01, 90% relaxed and 95% strict confidence level, and accounted for relatedness of candidate mothers and fathers. Relatedness was calculated with the R package related 1.0^89^ using Wang’s estimator *r*^90^. This estimator was chosen because it performed best in simulations, showing the highest correlation between observed and expected values for our set of loci. We also specified a proportion of candidate fathers sampled as 0.85 to allow for unsampled males in adjacent territories and potential floaters. Additionally, we used Colony 2.0.6.5^91^ to verify parentage assignments from Cervus. Unlike Cervus, Colony reconstructs a full pedigree, inferring sibship and parentage among individuals by comparing the likelihood of different clusters of individuals and maximizing group rather than pairwise likelihoods. For this analysis, we used an error rate of 0.01, male and female polygyny, and a sibship size prior of 1.6, calculated as the average number of offspring per family group in our study population.

For both Cervus and Colony analyses, the set of candidate fathers included all sampled adult males plus the oldest subadult male from Group 6 that had dispersed from his natal group in the beginning of the study and could have sired offspring by the end of the sampling period. The set of candidate mothers included all adult females that shared their mtDNA haplotype with candidate offspring. For seven offspring (Supplementary Table S1), the mothers were known because they were seen nursing them. To test the reliability of our parentage estimates, we ran the analyses twice, with and without the respective set of known mother–offspring pairs. Combined non-exclusion probability for the set of 16 autosomal loci (with chr01b excluded) was 9.9 × 10^−5^ for the first parent, 3.4 × 10^−7^ for the second parent, and 9.0 × 10^−12^ for the parent pair, calculated using Cervus.

#### (2) Relatedness-based mate choice

To test if titis avoid mating with related individuals, we compared relatedness between real and randomly generated mating partners using the pairwise relatedness estimator implemented in STORM^92^. First, STORM calculates the relatedness of real mating pairs using the estimator of Li ^93^, with each locus weighted using the method described in^94,95^. Then, the program calculates the expected relatedness of mating pairs if the mating is random with respect to relatedness; this is done by generating mating pairs from female and male breeding pools over 1000 iterations and averaging the relatedness values for each iteration. The obtained distribution is then compared to the averaged relatedness of real mating pairs. Our sample included ten real mating pairs, and the breeding pool consisted of 12 females and 12 males. This included all sampled adults and the oldest subadult male from Group 6.

#### (3) Heterozygosity-based mate choice

To test if titis show any heterozygosity-based mating pattern, we compared individual heterozygosity levels between pair mates. To estimate individual heterozygosity, we calculated homozygosity by loci (HL), ﻿a microsatellite-derived measure that weights the contribution of each locus to the homozygosity value depending on their allelic variability, implemented in R function GENHET 3.1^96^. To test if HL is correlated between pair mates, we used a two-tailed Pearson correlation analysis.

#### (4) Dispersal patterns

To examine if dispersal distances differ between sexes, we compared the diversity of mtDNA haplotypes, relatedness, and heterozygosity among adult females and males. MtDNA haplotype and nucleotide diversity was calculated and compared using a permutation test implemented in R function genetic_diversity_diff 1.0.6 (^97^; available from https://github.com/laninsky/genetic_diversity_diffs). We included 12 sampled adult females and 12 adult males in this analysis, plus two females that could not be sampled but whose haplotypes were inferred from the haplotypes of their offspring (the adult female of Group 4, who supposedly had been replaced before the study period, and the adult female of Group 9, who was present during the study period but could not be sampled). Relatedness among females and among males was calculated using Wang’s estimator *r* and then compared using 1000 bootstrapping samples in Coancestry 1.0.1.9^98^. In this analysis, as well as in the tests described below, we included 12 sampled adult females and 12 males. Individual heterozygosity was calculated using HL estimator (homozygosity by locus, see above) and compared between sexes using a paired t-test.

To evaluate spatial genetic structure, we conducted a spatial autocorrelation analysis following Smouse and Peakall^99^ in PopGenReport 2.2.2^86^, separately for adult females and males. The analysis calculated the correlation coefficient *r* between pairwise genetic distances, calculated using microsatellite genotypes with the method of Smouse and Peakall^99^, and pairwise spatial distances, for each distance class. The coefficient *r* is bound between -1 and 1 and has a mean of zero when there is no correlation. As a measure of spatial distances, we used distances between centroids of home ranges calculated with ArcGIS Desktop 10.6 (ESRI). These distances varied from 215 to 3200 m.

To further evaluate spatial genetic structure in females, we conducted a test similar to spatial autocorrelation analysis using mtDNA haplotype distances, correlating the number of nucleotide differences between haplotypes with spatial distances. For this test, if a spatial genetic structure is present, a positive correlation between haplotype and spatial distances is expected. We used Mantel tests with 10,000 permutations in R package ecodist^100^.

### Ethical approval

This work was conducted under all necessary permits (Research Permit No. 249-2017-SERFOR/DGGSPFFS from the Servicio Nacional Forestal y de Fauna Silvestre of the Peruvian Ministry of Agriculture) and ethical guidelines from the relevant authorities of Peru and the German Primate Center.

## Supplementary information

Supplementary Information 1.

Supplementary Information 2.

Supplementary Information 3.

## Data Availability

All data needed to evaluate the conclusions in the paper, including genotypes of all study animals, are present in the paper and/or the Supplementary Materials. Additional data related to this paper may be requested from S.D. (s.dolotovskaya@gmail.com) or C.R. (croos@dpz.eu).
