# Supplementary material for: Genetic monogamy and mate choice in a pair-living primate
Source: Sci Rep. 2020 Nov 23;10:20328. doi: 10.1038/s41598-020-77132-9 (PMC7683532; doi:10.1038/s41598-020-77132-9)
Supplement: Supplementary file 2 — Supplementary Information 2. [file 41598_2020_77132_MOESM2_ESM.pdf]

Genetic monogamy and mate choice in a pair-living primate

Sofya Dolotovskaya[1, 2*], Christian Roos[2,3,§], Eckhard W. Heymann[1, §]

[1] Behavioral Ecology and Sociobiology Unit, German Primate Center, Göttingen, Germany

[2] Primate Genetics Laboratory, German Primate Center, Göttingen, Germany

[3] Gene Bank of Primates, German Primate Center, Göttingen, Germany

[§] CR and EWH share senior authorship

* Corresponding author. Email s.dolotovskaya@gmail.com

Spatial autocorrelation analysis: supplementary results

Table S2. Results of spatial autocorrelation analysis following Smouse and Peakall [1] for females and males separately. Shown are the maximum pairwise geographic distances for each distance class, correlation coefficient *r*, the number of animals in each distance class and lower (r.l) and upper (r.u) bounds for the 95% confidence interval of *r*, obtained by bootstrapping.

**Females**					**Males**				
Distance class, m	N animals	r	r.l	r.u	Distance class, m	N animals	r	r.l	r.u
299.4	6	-0.0830325	-0.2542848	0.21937966	292.8	4	-0.2349362	-0.3567092	0.13736895
598.8	18	-0.0981039	-0.2324864	0.02939302	585.6	14	-0.1058502	-0.2257818	0.01909891
898.2	36	-0.0773378	-0.1769578	-0.0002104	878.4	22	-0.1169571	-0.2111225	0.01247317
1197.6	26	-0.0978059	-0.1831366	0.03261151	1171.2	14	-0.0903676	-0.2124641	0.00493281
1497	12	-0.1212871	-0.240429	0.07607147	1464	12	-0.068062	-0.2449801	0.0480202
1796.4	10	-0.063905	-0.2753533	0.08319532	1756.8	6	-0.104962	-0.2914423	0.18519218
2095.8	0	NA	NA	NA	2049.6	4	-0.029389	-0.2991212	0.24345271

2395.2	8	-0.1285922	-0.2708487	0.14497517	2342.4	12	-0.0757605	-0.2306265	0.04671697
2694.6	6	-0.1145511	-0.2889671	0.11391038	2635.2	8	-0.0743862	-0.2061112	0.07836299
2994	4	-0.0106682	-0.3831919	0.1271796	2928	8	-0.124718	-0.22446	0.08496394

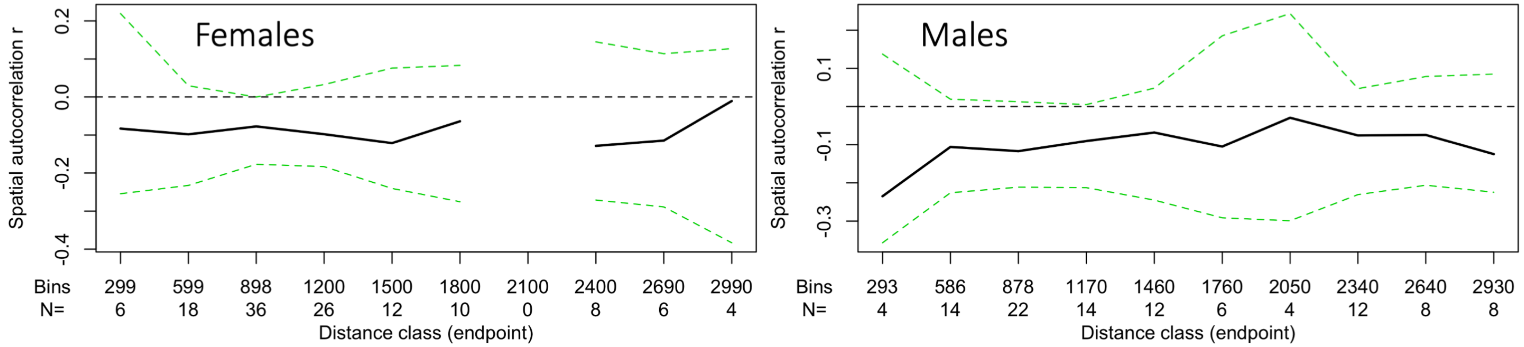

Fig. S1. Autocorrelation coefficient *r*, calculated following Smouse and Peakall [1] for females (A) and males (B) separately, for each distance class. Dotted line represents lower and upper bounds for the 95% confidence interval of *r*, obtained by bootstrapping.

Microsatellite genotyping: supplementary methods

As published microsatellite loci for titi monkeys [2–4] revealed unreliable results for our study species, we established a new set of 27 di-repeat microsatellite loci that can be applied not only for our study species but for all New World monkeys (Tables S3–S4). Therefore, we screened 450 bacterial artificial chromosome (BAC) clones of *Plecturocebus moloch* (as representative of the Pitheciidae and thus most basal family among New World monkeys) available in Genbank for microsatellites using RepeatMasker (http://www.repeatmasker.org) and extracted orthologous regions (microsatellite plus 1000 bp flanking regions) from the four available New World monkey genomes (*Callithrix jacchus* calJac3/ASM275486v1, *Saimiri boliviensis boliviensis* SaiBol1.0, *Cebus capucinus imitator* Cebus_imitator-1.0, *Aotus nancymaae* Anan_2.0) available at the UCSC Genome Browser (https://genome.ucsc.edu) and the Ensemble webpage (https://www.ensembl.org/index.html). Only loci with length variation in the microsatellite repeat among the five New World monkey species were selected for further analysis. Further selection criteria were: 1) loci are on

different chromosomes according to the *Callithrix jacchus* genome, 2) primers bind in conserved flanking regions of the microsatellite, 3) primers do not bind in repeat regions such as Alu elements, and 4) primers are close to the microsatellite to minimize amplicon size, thus allowing amplification from low-quality DNA samples. In total 27 loci fulfilled these criteria. Primers were designed to carry adapter nucleotide sequences at their 5' ends (forward primer: 5'-ACACTCTTTCCCTACACGACGCTCT-3'; reverse primer: 5'-GTGACTGGAGTTCAGACGTGTGCTCTTCCGATCT-3') to simplify library preparation for genotyping by sequencing on Illumina's MiSeq desktop sequencer.

We then tested if the primers are locus-specific and if these loci can be universally amplified across New World monkeys by conducting singleplex PCRs with each one representative of all New World monkey families and subfamilies (Pitheciidae-Callicebinae: *Plecturocebus cupreus,* Pitheciidae-Pitheciinae: *Chiropotes satanas,* Atelidae-Alouattinae: *Alouatta seniculus,* Atelidae-Atelinae: *Ateles fusciceps,* Aotidae: *Aotus azarae,* Cebidae-Cebinae: *Sapajus xanthosternos,* Cebidae-Saimirinae: *Saimiri boliviensis,* Callithrichidae: *Callimico goeldii*). Therefore, we used high-quality DNA extracted from blood or tissue samples available in the Gene Bank of Primates at the German Primate Center. PCR reactions were performed with the Qiagen Multiplex PCR Kit (Qiagen) in a total volume of 25 μL and containing 12.5 μL 2x Multiplex Master Mix, 0.5 μL (0.2 μM) of each primer, 1 μL (ca. 20 ng) of DNA and 10.5 μL of RNase-free water. Amplifications were performed with initial denaturation at 95°C for 15 min, 40 cycles of denaturation at 94°C for 30 sec, annealing at 57 °C for 1.5 min, extension at 72 °C for 1 min and a final extension at 60°C for 30 min. PCR products were checked on 1.5% agarose gels together with non-template controls and size standards.

Next, we checked if all 27 loci can also be amplified in a single multiplex PCR reaction. Therefore, we again used the eight high-quality DNA extracts and applied the methods (PCR amplification, gel electrophoresis, purification steps, indexing PCR, sequencing on MiSeq platform, data analysis with CHIIMP software) as outlined in the main text. We also checked if the amplification would be more successful when using 3 separate multiplex reactions with the following primer pools: chr01b–chr07a, chr08a–chr12a, chr12b–chrXa (the order is the same as in table S3; reaction details are provided in the main text). The results of these runs, both for the total and 3 separate multiplex reactions, are provided in table S3. Loci chr11f and chr16b failed to amplify in all samples, and loci chr96a and chr06b were amplified in less than half of the samples; the rest of the loci amplified in at least half of the samples. The number of successfully amplified loci varied from 16 (*Callimico goeldii* and *Chiropotes satanas*) to 25 (*Sapajus*

xanthosternos, *Saimiri boliviensis*). Using 3 separate multiplex PCR reactions in most cases yielded more reads than using one total multiplex reaction; the alleles called, however, did not differ between these methods.

Table S4. Information on the 27 microsatellite loci designed in this study: locus name, forward and reverse primer sequences, location in the *Callithrix jacchus* genome, repeat motif, size range and BAC clone accession number.

**Locus**	**Forward primer**	**Reverse primer**	**Location CalJac3**	**Repeat motif**	**Size range, bp**	**PMOL BAC clone Acc. Nr.**
PMOL_chr01b	TGCCAAGGGCTTTCTGA	AAACAYCTCTTTTGTAGAAG	chr01:195360092-195360177; 1q	TG	75-105	AC187426.3
PMOL_chr02a	AGGCTGTGTTTGTGGTG	TTCTTGCACCTTTCTCAAT	chr02:47304050-47304166; 2p	TG	117-123	AC188274.1
PMOL_chr02b	GGTCAAACCAGGGCAAA	TTATTTGCAATTTATAGCCTA	chr02:72990959-72991145; 2q	GC, AC	139-187	AC186467.2
PMOL_chr03e	GTAAGATGGGAGATTAGC	ATTACAGCCCTATGGTAG	chr03:167338029-167338166; 3q	TG	112-138	AC188271.1
PMOL_chr04a	AAGAAAAGTGAGATCCCC	TGTTGAGGTTGCCCAGA	chr04:42663591-42663697; 4q	TG	97-131	AC187951.2
PMOL_chr05c	GCCCCACACCTGCTTT	GACCACCTGCCACATG	chr05:9622769-9622890; 5p	TG	102-145	AC200393.3
PMOL_chr05g	TTTTGAATCCTTTCCAGTG	ATTCCTGAGCTCAGGTTT	chr05:1808047-1808162; 5p	CA	112-122	AC187427.2
PMOL_chr06a	ATGGAACAGCCAATGAGA	TYTTAAGTAGAGGAGTGAC	chr06:91748983-91749061; 6q	CT	78-104	AC209150.1
PMOL_chr06b	AGCTGTGAACATTTTGTAC	AATTACAGCATATTCATGCT	chr06:12239728-12239857; 6p	CA, CT	120-142	AC157438.1
PMOL_chr07a	TGCCTGAGAACTGCACA	GACATGCTTTCCCTCAAT	chr07:60957721-60957833; 7q	TG, CG	106-134	AC188276.1
PMOL_chr08a	GTAGAGCTAAGAGGCTC	TCATTTAAGAATAGGCAATG	chr08:84171068-84171190; 8q	TG, GC	87-123	AC203507.2
PMOL_chr08f	GGTAGTTGTTGGCACTG	ACATGATATATAAGGGGAG	chr08:119173148-119173258; 8q	AC, GT, GA	91-123	AC174857.2
PMOL_chr09a	GTTCTGCCTTAAGGTTTC	CATAAARATCCACTTTAAAAC	chr09:10544128-10544250; 9p	TG	111-125	AC244997.1
PMOL_chr10a	GTGCAGGGACAAATCTG	TGGCCTTGTAAATAAAATGT	chr10:125088776-125088873; 10q	CA	90-110	AC188357.2
PMOL_chr10b	AGAAGCCATGTCAATTAAG	ATTGTCAAAATATGGCTCC	chr10:20581481-20581580; 10q	TG	86-114	AC186463.2
PMOL_chr11e	CCTGGGCTTACAGAACC	TACCTATCTAGCTCATTTC	chr11:14130663-14130767; 11p	CA	105-122	AC186115.2
PMOL_chr11f	TATGCAATATATTTCAAATATC	GATGCTGATGCATTTGTG	chr11:68860458-68860555; 11q	CA	92-112	AC172721.2
PMOL_chr12a	AGTGACTGTTTAACCACC	TCCATTTTACAACTGCTGA	chr12:24867917-24868037; 12p	TG	107-147	AC193735.2
PMOL_chr12b	CAAATATAGACTCATTAAATG	CTACAGGTATGTTCCTTG	chr12:70126419-70126506; 12q	AC	88-110	AC190366.1
PMOL_chr13a	GCTCAGACAGGATGATG	AGAGCTTTGGACTCAGG	chr13:101919673-101919801; 13q	CA	95-129	AC187952.2

PMOL_chr13b	GCAAGAGTGGTCTGGC	CAAGAATTATCTATGCAGG	chr13:6811774-6811922; 13p	CA	131-161	AC151890.2
PMOL_chr14b	TTAGGCATTGATATAAGGC	CAGAAAAATTTCATTGCCC	chr14:10607938-10608090; 14	CA, TA, GA	143-189	AC207510.1
PMOL_chr16a	AGCACATGACTGGCCTT	AAGATAACAAATAGAATTGGA	chr16:76914251-76914383; 16	CA	117-133	AC190368.2
PMOL_chr16b	CTGCAGACTAGCCTCAT	GGATTTACAAAGGAAATAGA	chr16_GL285730_random:61519-61625; 16	TG, AG	107-133	AC186936.2
PMOL_chr18a	AGCTGGTTTGGGAGATAA	TGCTCAGATYCTCAGTCT	chr18:5739039-5739139; 18	AC	70-101	AC189181.2
PMOL_chr21a	GAATTTCTTCAGTTCAACTA	CRGTGTTAAGATTGAAAATG	chr21:37574331-37574478; 21q	GA, TG	130-148	AC174416.2
PMOL_chrXa	ATGTGTTGTGGACCTAAG	TCCAAGAAGTAATCGTGTA	chrX:125242408-125242500; Xq	AC	93-117	AC237129.1

Table S5. Information on the 18 microsatellite loci used in the study population of *Plecturocebus cupreus*: locus name, repeat motif, size range, number of alleles, observed (HO) and expected (HE) heterozygosity estimated in PopGenReport, p-value of the Hardi-Weinberg equilibrium test (HWE) (with Bonferroni adjustment p < (0.05/ 17) = 0.00294 for autosomal loci and p < (0.05/ 18) = 0.00278 for chrXa; significant deviations marked in bold) as calculated in PopGenReport, null allele frequencies estimated using Brookfield1 method in MicroChecker and number of individuals typed for each locus.

**Locus**	**FullName**	**Repeat motif**	**Size range, bp**	**Number of alleles**	**HO**	**HE**	**HWE-P**	**Null F**	**N ind typed**
1	chr01b	TG	89–123	12	0.609	0.905	**0.000**	0.072	28
4	chr03e	AC	106–138	12	0.826	0.889	0.020	-0.013	39
6	chr05c	AC	103–125	9	0.739	0.742	0.730	-0.022	40
7	chr05g	TG	102–116	8	0.913	0.828	0.747	-0.056	41
8	chr06a	GA	90–119	12	0.870	0.857	0.525	-0.046	41
10	chr07a	CA	106–122	8	0.913	0.805	0.119	-0.048	39
11	chr08a	AC	86–93	6	0.826	0.757	0.162	-0.072	40
12	chr08f	AC	109–143	11	0.913	0.874	0.039	0.039	36

13	chr09a	CA	107–129	8	0.870	0.782	0.173	-0.050	41
14	chr10a	GT	94–114	9	0.783	0.840	0.742	0.059	41
16	chr11e	GT	90–124	9	0.696	0.775	0.514	0.060	41
19	chr12b	TG	74–90	3	0.565	0.466	0.835	-0.002	38
20	chr13a	GT	101–106	4	0.348	0.502	0.099	-0.001	41
22	chr14b	TG	147–173	14	0.826	0.897	0.078	0.001	39
23	chr16a	GT	113–119	4	0.174	0.309	0.011	0.027	39
25	chr18a	GT	78–124	16	0.826	0.912	0.034	-0.007	38
26	chr21a	GA	157–161	5	0.696	0.716	**0.000**	-0.117	34
27	chrXa	TG	105–127	10	0.872	1.000	0.816*	-0.068	19

Direct observation of dispersal

In September 2018, we witnessed the dispersal of a subadult male (Snape), the oldest offspring of Group 1, from its natal home range and the formation of a new pair. Group 1 was seen feeding every day during a 2-week period in an *Inga edulis* tree next to the station buildings. On all occasions, all group members were feeding simultaneously and without any aggression. On September 8, the adult female was aggressively chasing the young adult male while both were leaving the feeding tree. The next day, the group was not seen feeding, and on September 10, the young adult male was feeding alone. On the early morning of September 11, the group was heard calling south-east from the station, and a single individual, presumably the young adult male, was heard from the south-west. On 12 September, the young adult male was encountered in the company of an unknown female south-west of the station. They established in an unoccupied area between the home ranges of Group 1 and Group 6 (Fig. 1 in the main text). Subsequently, this newly formed pair (Group 11) was having intergroup encounters with Group 1 almost every morning; calling and counter-calling were accompanied occasionally by some chasings between the newly formed pair and the adult male from Group 1. Calling and counter-calling ceased by around December 1, 2018. As of September 2019, Group 11 was still occupying the same home range and had an infant born in February 2019.

References

1. Smouse, P. E. & Peakall, R. Spatial autocorrelation analysis of individual multiallele and multilocus genetic structure. *Heredity (Edinb).* **82**, 561–573 (1999).
2. Mendoza, A. *et al.* Population genetics of the California National Primate Research Center's (CNPRC) captive *Callicebus cupreus* colony. *Primates* **56**, 37–44 (2015).
3. Menescal, L. A., Gonçalves, E. C., Silva, A., Ferrari, S. F. & Schneider, M. P. C. Genetic diversity of red-bellied titis (*Callicebus moloch*) from Eastern Amazonia based on microsatellite markers. *Biochem. Genet.* **47**, 235–240 (2009).
4. Martins, A. B. Characterization and evaluation of microsatellite loci suitable for studies on mating system, parentage, and genetic identity in red titi monkeys (*Callicebus discolor*) and saki monkeys (*Pithecia aequatorialis*). (The University of Texas at Austin, 2015).
